# Occurrence of Carcinoma of the Pancreas Following Nilotinib Therapy for Chronic Myeloid Leukemia: Report of a Case with Review of the Literature

**DOI:** 10.4274/tjh.2013.0322

**Published:** 2015-08-01

**Authors:** Yasunobu Sekiguchi, Asami Shimada, Moe Matsuzawa, Hidenori Imai, Mutsumi Wakabayashi, Keiji Sugimoto, Noriko Nakamura, Tomohiro Sawada, Junichi Arita, Norio Komatsu, Masaaki Noguchi

**Affiliations:** 1 Juntendo University Faculty of Medicine, Urayasu Hospital, Clinic of Hematology, Urayasu, Japan; 2 Juntendo University Faculty of Medicine, Urayasu Hospital, Clinic of Clinical Laboratory, Urayasu, Japan; 3 The Cancer Institute of the Japanese Foundation for Cancer Research, Gastroenterology Center, Tokyo, Japan; 4 Juntendo University Faculty of Medicine Hospital, Department of Hematology, Tokyo, Japan

**Keywords:** Chronic myeloid leukemia, Nilotinib, Secondary malignancy, Carcinoma of the pancreas

## Abstract

The patient, a 79-year-old Japanese man, was diagnosed with the chronic phase of chronic myeloid leukemia and begun on nilotinib therapy in April 2011. The therapeutic response was major molecular response in August. About 19 months after the start of nilotinib therapy at 400 mg/day (November 2012), an adenocarcinoma (24x20 mm) confined to the head of the pancreas developed. In February 2013, a pancreaticoduodenectomy was performed. The therapy regimen was switched to dasatinib at 100 mg/day, beginning in April. The response was still major molecular response with no recurrence of pancreatic carcinoma in July 2013. There have been 29 reported cases of secondary neoplasms associated with nilotinib therapy. These secondary neoplasms were characterized by relatively frequent occurrence of papilloma (6 cases), gastric cancer (3 cases), fibroma (3 cases), and thyroid neoplasms (2 cases). The present case, however, is the first to be reported as carcinoma of the pancreas. This report describes the case.

## Abstract (tr)

Yetmiş dokuz yaşında Japon erkek hastaya, Nisan 2011’de kronik evre kronik miyeloid lösemi tanısı konulup, nilotinib tedavisi başlandı. Ağustos’ta majör moleküler yanıt sağlandı. Nilotinib 400 mg/gün dozunda kullanılırken ve tedavi başlangıcından yaklaşık 19 ay geçmişken (Kasım 2012’de), pankreas başına sınırlı (24x20 mm) adenokanser gelişti. Şubat 2013’te pankreatikoduodenektomi yapıldı. Nisan ayından başlayarak tedavi rejimi olarak dasatinib 100 mg/gün’e geçildi. Temmuz 2013’te yanıt halen majör moleküler yanıttı ve pankreas kanserine ait nüks saptanmadı. Literatürde nilotinib tedavisi ile ilişkili olan 29 ikincil neoplazi tespit edildi. Bu ikincil neoplaziler arasında görece daha sık gözlemlenenler papilloma (6 olgu), mide kanseri (3 olgu), fibroma (3 olgu) ve tiroid neoplazileridir (2 olgu). Olgumuz, literatürde nilotinib altında iken gelişen ilk pankreas kanseri olgusudur. Bu yazıda olgu tartışması ve nilotinibe bağlı gelişen ikincil kanserlere ait literatür taraması sunulmaktadır.

## INTRODUCTION

Regarding secondary neoplasms (SNs) during imatinib therapy, a previous report described that there was no increase in incidence, while another report stated that an approximately 1.5-fold increase, though not statistically significant, was noted for patients as compared to incidence for a general population [1,2]. It has also been pointed out in a few reports that the incidence of certain types of malignant tumors has increased. An increased incidence has been documented for carcinoma of the prostate; melanoma, carcinoma of the kidney, endocrinoma, and chronic lymphoid leukemia; cancer of the prostate and breast cancer; and non-Hodgkin lymphoma and cancers of the gastrointestinal tract, lungs, and skin [3,4,5,6]. Further, SNs in patients with chronic myeloid leukemia (CML) are complicated and have been described to be probably ascribed to multiple factors [7,8]. Our extensive search of the literature revealed the time to onset of SN after start of nilotinib therapy to be unclear but that after start of imatinib therapy to be about 39 months [4].

There have been no more than a total of 29 cases of SNs associated with nilotinib therapy reported, including the present case [4,6,9,10,11,12]. As second-generation tyrosine kinase inhibitors, including nilotinib, are more potent in immunosuppressive activity than imatinib, their potential risk of SN has been pointed out [4,13]. Long-term follow-up of accumulated cases treated with nilotinib, as is the case with imatinib, is considered necessary.

Pancreatitis occurring during nilotinib therapy was noted with an incidence of 1.9%, being higher as compared to imatinib and dasatinib, and it tended to be more frequent among Japanese patients [10,14,15,16]. Since chronic pancreatitis represents a risk factor for cancer of the pancreas, nilotinib may possibly constitute a risk factor for pancreatic carcinoma [17]. The present case is the first to be reported as pancreatic carcinoma that developed following nilotinib therapy.

## CASE PRESENTATION

**Figure 1:**
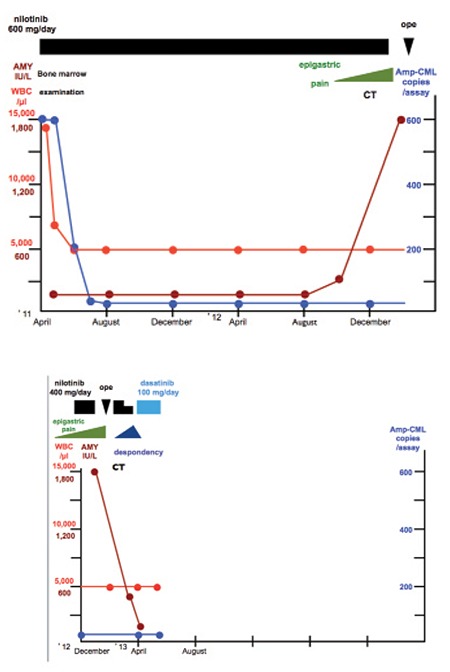
Clinical course: A diagnosis of chronic phase of chronic myeloid leukemia was made and the patient was begun on nilotinib at 400 mg/day. He obtained major molecular response. He began suffering from postprandial epigastric pain and a computed tomography scan revealed a tumor mass in the head of the pancreas. The mass was diagnosed as an adenocarcinoma. Nilotinib was discontinued and a pancreaticoduodenectomy was performed. Nilotinib at 400 mg/day was reinstituted, but this was switched to dasatinib at 100 mg/day when despondency appeared. The listless feeling then disappeared and the response is still major molecular response with no indication of pancreatic cancer recurrence.

*Patient:* A Japanese man aged 79, with a chief complaint of increased leukocyte and platelet counts. Past history: He had experienced hepatic angioma and lumbar disk herniation, had an operation for acute appendicitis, was operated on for chronic sinusitis, and had been known to be hypertensive with benign prostatic hypertrophy. Family history, life history, and medication history: Unremarkable. Present illness: The patient had been receiving doxazosin mesylate and tamsulosin hydrochloride since 2003 for hypertension and benign prostatic hypertrophy at a nearby medical clinic. He was referred to us for medical workup in April 2011 because of increased leukocyte count (12,900/µL) and platelet count (69.8x104/µL). Laboratory findings: See Table 1. The amylase (AMY), carbohydrate antigen 19-9 (CA19-9), s-pancreas-1 antigen (SPAN-1), and pancreatic cancer-associated antigen (DUPAN-2) levels were all within their respective reference ranges. Bone marrow examination revealed a nucleated cell count of 36.0x104/µL, a megakaryocyte count of 915/µL, 1.4% blasts, and chromosomal aberrations 46,XY, t(9;22) (q34; q11.2). The patient was thus diagnosed with the chronic phase of CML and was begun on nilotinib at 400 mg/day in April 2011 (Figure 1). The dose was reduced to 400 mg/day because the patient was elderly. In August 2011, i.e. 4 months later, the peripheral blood fused gene level became Amp-CML <5 copies/assay, signaling a major molecular response (MMR) [18].

**Figure 2:**
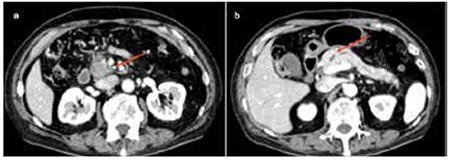
Computed tomography scans of the abdomen: (a) A 24x20-mm tumor mass was noted in the head of the pancreas (red arrow). (b) The main pancreatic duct was dilated, but there was neither vascular abnormality nor lymph node swelling (red arrow).

In November 2012, the patient began suffering from postprandial epigastric pain, and the following parameters were noted to be elevated: AMY: 326 IU/L, CA19-9: 38.2 U/mL, SPAN-1: 33 U/mL, and DUPAN-2: >1600 U/mL. A computed tomography (CT) scan disclosed a mass measuring 24x20 mm in the head of the pancreas but there was neither vascular abnormality nor any lymphadenopathy (Figure 2). A diagnosis of adenocarcinoma was made from needle biopsy findings of the mass in the head of the pancreas. Nilotinib was discontinued late in January 2013, and the patient had a pancreaticoduodenectomy elsewhere in the middle of February. The postoperative course was uneventful. Informed consent was obtained.

In March, treatment with nilotinib at 400 mg/day was resumed, at which time the response was still MMR. However, despondency appeared from immediately after reinstitution of nilotinib, which was therefore then switched to dasatinib at 100 mg/day in April. The listless feeling disappeared and the patient progressed favorably with continued oral dasatinib. The response is still MMR and there was no recurrence of cancer of the pancreas as of July 2013.

## DISCUSSION AND REVIEW OF THE LITERATURE

Twenty-nine cases of SN have been reported in the literature to our knowledge so far as nilotinib is concerned: 9 cases in global phase III multicenter trials, 2 cases in a Japanese phase II study, 4 cases in an overseas phase II trial, 4 cases reported by Verma et al. (3 patients receiving nilotinib switched from imatinib, and 1 patient on nilotinib as a first-line regimen), 7 cases in postmarketing clinical use surveys in Japan, and the case documented herein (Table 2) [4,9,10,11,12]. The time to onset of SN after initiation of nilotinib therapy was unclear. Of the SNs reported, papilloma in 6 cases, gastric cancer in 3 cases, fibroma in 3 cases, and thyroid neoplasms in 2 cases were relatively common, while the present case is the first to be reported as carcinoma of the pancreas. Nilotinib was given as an up-front treatment in some instances or as a second-line treatment in others. Therefore, the impact of these differences, if any, on the occurrence of secondary neoplasms remains uncertain.

It was also reported that an increase in the incidence of the following neoplastic changes was noted in a rat 2-year carcinogenicity study of imatinib: adenoma/adenocarcinoma of the kidney, urinary tract papilloma, adenocarcinoma of the small intestine, adenoma of the parathyroid, adrenal benign and malignant medullary tumors, papilloma/squamous cell carcinoma of the forestomach, papilloma of the clitoral gland, squamous cell carcinoma of the preputial gland, and papilloma of the preputial gland [15]. In a 2-year carcinogenicity study of dasatinib in rats, the incidence of papilloma and squamous cell carcinoma of the uterus and adenoma and adenocarcinoma of the prostate was observed to be increased at dose levels equal to or even lower than a clinical exposure level [16]. Thus, the potential risk of secondary malignancies cannot be ruled out for nilotinib, although there are no animal experimental data available at present. Verification by animal experiment would be needed. Inasmuch as nilotinib and dasatinib have greater immunosuppressive potency than imatinib, there has been a report pointing out the risk of SNs; therefore, long-term follow-up of accumulated cases treated with nilotinib, as is the case with imatinib, is considered necessary [4,13].

Pancreatitis occurring during nilotinib therapy was reported in 1.9% of patients, and this incidence rate is higher as compared to less than 1% for imatinib, while it is unknown for dasatinib [14,15,16]. It is assumed that nilotinib acts upon an unknown intracellular pathway involved in calcium release from pancreatic acini, thereby regulating pancreatic exocrine secretion, and inhibits acinar opening/exocrine secretion by facilitating fatty acid deposition in pancreatic acinar cells, yet the underlying details remain to be clarified [19]. Thus, there is the possibility that nilotinib may be a risk factor for pancreatitis. Increased plasma lipase level was noted in 23.5% of patients treated in a Japanese phase II study, this incidence being particularly high as compared to 8.3% in global phase III multicenter trials and 12.9% in an overseas phase II trial (Table 3) [9,10,11]. There may be an interracial difference in this respect. Because chronic pancreatitis represents a risk factor for cancer of the pancreas, the possibility that nilotinib eventually may constitute a risk factor for pancreatic carcinoma and that Japanese race may also be a risk factor cannot be negated [17]. The patient reported herein had no history of pancreatitis. The present case is the first to be documented as carcinoma of the pancreas that developed following nilotinib therapy. Long-term outcome data analysis based on careful follow-up observation including periodic pancreatic enzyme checkups and by-race classification of study population is considered to be needed.

## Figures and Tables

**Table 1:**
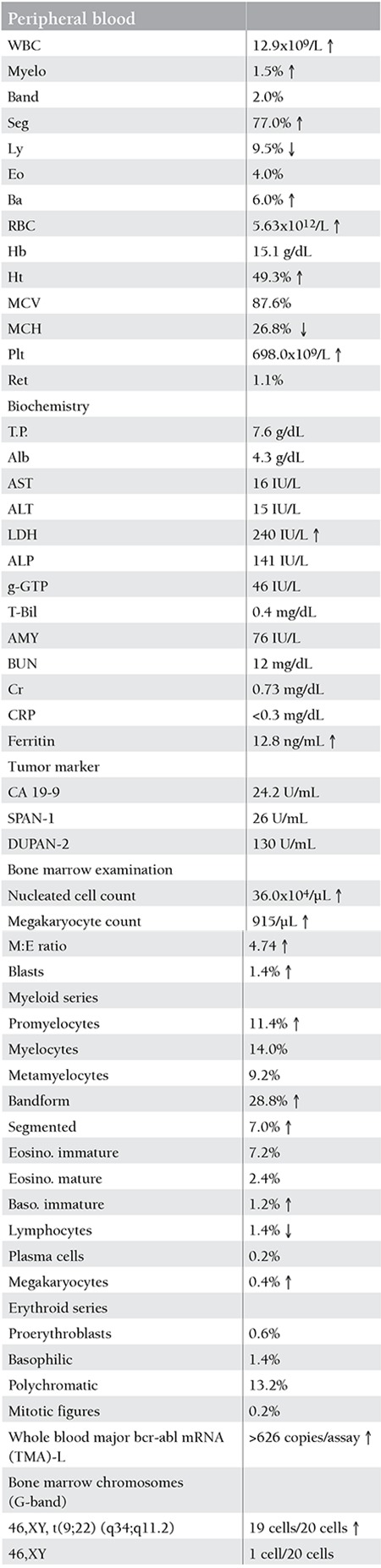
Laboratory findings upon initial examination in this department.

**Table 2:**
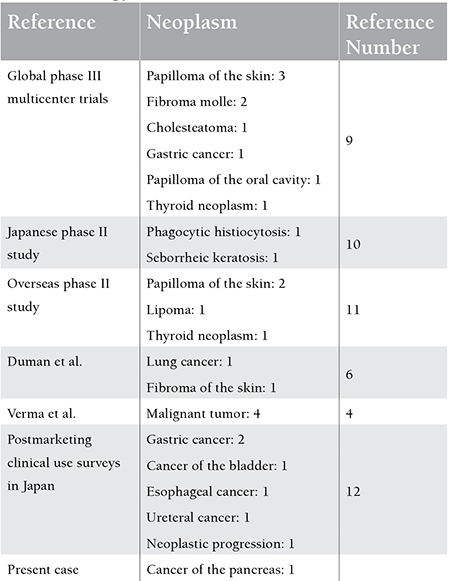
Reported secondary neoplasms associated with nilotinib therapy.

**Table 3:**
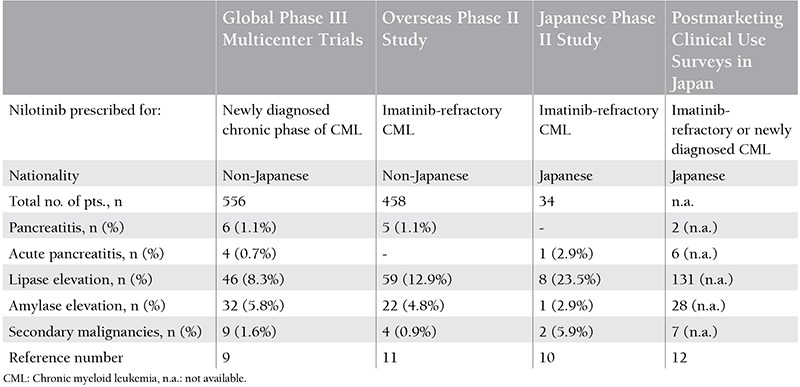
Reports of pancreatitis, pancreatic enzyme elevation, and secondary malignancies associated with nilotinib therapy.
